# Main N6-Methyladenosine Readers: YTH Family Proteins in Cancers

**DOI:** 10.3389/fonc.2021.635329

**Published:** 2021-04-13

**Authors:** Xin-Yuan Dai, Liang Shi, Zhi Li, Hai-Yan Yang, Ji-Fu Wei, Qiang Ding

**Affiliations:** ^1^Jiangsu Breast Disease Center, The First Affiliated Hospital With Nanjing Medical University, Nanjing, China; ^2^Research Division of Clinical Pharmacology, The First Affiliated Hospital With Nanjing Medical University, Nanjing, China

**Keywords:** cancer, IYT521-B homology domain proteins, N6-methyladenosine, cancer therapy, RNA metabolism

## Abstract

Among the over 150 RNA modifications, N6-methyladenosine (m6A) is the most abundant internal modification in eukaryotic RNAs, not only in messenger RNAs, but also in microRNAs and long non-coding RNAs. It is a dynamic and reversible process in mammalian cells, which is installed by “writers,” consisting of METTL3, METTL14, WTAP, RBM15/15B, and KIAA1429 and removed by “erasers,” including FTO and ALKBH5. Moreover, m6A modification is recognized by “readers,” which play the key role in executing m6A functions. IYT521-B homology (YTH) family proteins are the first identified m6A reader proteins. They were reported to participate in cancer tumorigenesis and development through regulating the metabolism of targeted RNAs, including RNA splicing, RNA export, translation, and degradation. There are many reviews about function of m6A and its role in various diseases. However, reviews only focusing on m6A readers, especially YTH family proteins are few. In this review, we systematically summarize the recent advances in structure and biological function of YTH family proteins, and their roles in human cancer and potential application in cancer therapy.

## Introduction

In recent years, post-transcriptional modification, which plays an important role in physiological and disease progression, has attracted more and more attention in molecular biology research. RNA modification is an emerging area of post-transcriptional modification. Among the over 150 RNA modifications, N6-methyladenosine (m6A) is identified as the most abundant and evolutionarily conserved internal RNA modification in eukaryotic RNAs, not only in messenger RNAs (mRNAs) (1), but also in microRNAs (2) and long non-coding RNAs (lncRNAs) (3). Although m6A was first discovered in the 1970s (1), the precise function of m6A residues in the regulation of gene expression has been identified until recent years with the advance of high-throughput sequencing technology, which mapped of m6A residues in the whole transcriptome (4, 5). Since then, numerous studies have carried out to verify the significance of m6A modification, which largely broadens the study in RNA fields (6, 7).

The m6A modification is mainly located in a consensus RNA motif of RRACH (R=A or G, H=A, U, or C) and particularly enriched in 3′ untranslated regions (3′ UTRs) and near stop codons (8). Similar to other epigenetic modifications like DNA and histone modification, m6A modification is dynamic and reversible. It is installed by “writers”, which refer to m6A methylase complex consisting of methyltransferase-like 3 (METTL3), methyltransferase-like 14 (METTL14), Wilms tumor 1 associated protein (WTAP), RNA binding motif 15/15B (RBM15/15B), and KIAA1429 (9–12). “Erasers” are m6A demethylases, including fat mass and obesity-associated protein (FTO) and AlkB homologue 5 (ALKBH5), which could remove m6A modification from RNAs and make m6A modification in a dynamic balance (13, 14). “Readers” refer to proteins that are capable to recognize and bind m6A modification, which mainly mediate the regulation of m6A modification in gene expression by affecting the fate of targeted RNAs (15). Reader proteins play the key role in executing m6A functions.

YT521-B homology (YTH) family proteins were the first identified m6A reader proteins. YTH family proteins can recognize m6A modification in a methylation-dependent manner through a specific YTH domain (16), which is recognized as a structured RNA binding domain and highly conserved among eukaryotes (17). By searching the human genome, there are five proteins in human genome carrying the YTH domain, which are divided into three categories: YTH m6A-binding protein (YTHDF) including YTHDF1-3, YTH domain-containing 1 (YTHDC1) and YTH domain-containing 2 (YTHDC2) (18). YTH family proteins are recruited by m6A and able to affect mRNA metabolism including mRNA splicing, nuclear export, translation, and mRNA degradation (6, 19). Accordingly, YTH family proteins are involved in many physiological processes, infection diseases and cancers. There are many reviews about the function of m6A and its role in various diseases (20, 21). However, reviews only focusing on m6A readers are few. In this review, we systematically introduce the main m6A readers YTH family proteins, summarize the structure features, the function of YTH family proteins in post-transcriptional gene regulation and their roles in tumorigenesis, cancer progression and potential application in cancer therapy. It may provide clues for the study of molecular mechanism of m6A readers in human cancers and exploring their clinical application in cancer treatment.

## Structure Features of YTH Domain

YTH domain was previously regarded was a structured RNA binding domain and highly conserved among eukaryotes (22). Subsequent studies proved that the ~150 amino acid YTH domain recognizes and binds RNAs in an m6A-dependent manner (16, 23). The structure of YTH family proteins have been understood until the crystal structures of the YTHDC1 YTH domain and YTHDC1–GG(m6A)CU complex were first studied, unveiling the molecular mechanism of m6A-YTH binding and sequence selectivity (16). The YTH domain adopts a conserved α/β fold containing 3α helices and 6β strands. 6β strands form a barrel fold and 3α helices are packed against the β strands to organize a hydrophobic core (16, 18). In the YTHDC1-GG(m6A)CU complex, the m6A is recognized by an aromatic cage consisting of 3 hydrophobic residues W377, W428, and L439. The aromatic cage of YTH domain forms a hydrophobic binding pocket that can recognize buried methyl groups with a cavity insertion mode. This complex structure provides the basis for specific m6A recognition by YTH domain. Compared to YTHDC1 YTH domain, other YTH family proteins have the similar cage structure. In YTHDC2, aromatic cage is composed of W1310, W1360 and L1365 (24); W411, W465 and W470 in YTHDF1 (25) and W432, W486 and W491 in YTHDF2 (26).

Despite conserved aromatic cages, YTH domain displays different sequence selectivity. The YTH domain of YTHDC1 favors a guanine nucleotide and disfavors an adenosine at the −1 position relative to the m6A. YTHDC1 shows preference to bind to the GG(m6A)C sequence. However, different from YTHDC1, YTHDF1, YTHDF2, and YTHDC2 YTH domains do not display sequence selectivity at the −1 position (25). The difference in sequence selectivity between YTHDC1 and other YTH family proteins may reflect the different m6A-binding requirement in the nucleus and cytoplasm (18). These structural data strongly support the proposal that YTH family proteins function as the reader for m6A.

Like most RNA binding domains which are surrounded by structured domains or low complexity regions with various functions (27), YTH domain is also flanked by several disordered regions. YTHDC1 is surrounded by regions rich in charged residues such as Glu-rich, Arg-rich segments, or in proline-rich (28). YTHDF family proteins are surrounded by Pro, Gln, and Asn-rich segments (26). Different from other YTH family proteins surrounded by low complexity regions, YTHDC2 is a multi-domain protein containing multiple helicase domains and two Ankyrin repeats. Consistent with its domain composition, YTHDC2 has ATPase and 3′→5′RNA helicase activities (29, 30). These unique features endow YTHDC2 with various functions, including recruiting and interacting with other protein complex members and exerting regulatory effects on RNA binding and RNA structure. These flanking regions are essential for YTH family proteins to exert functions in regulating fates of m6A-modified RNA by affecting the subcellular localization of YTH family proteins and their partners.

## Functions of YTH Family Proteins in mRNA Metabolism

**Figure 1:**
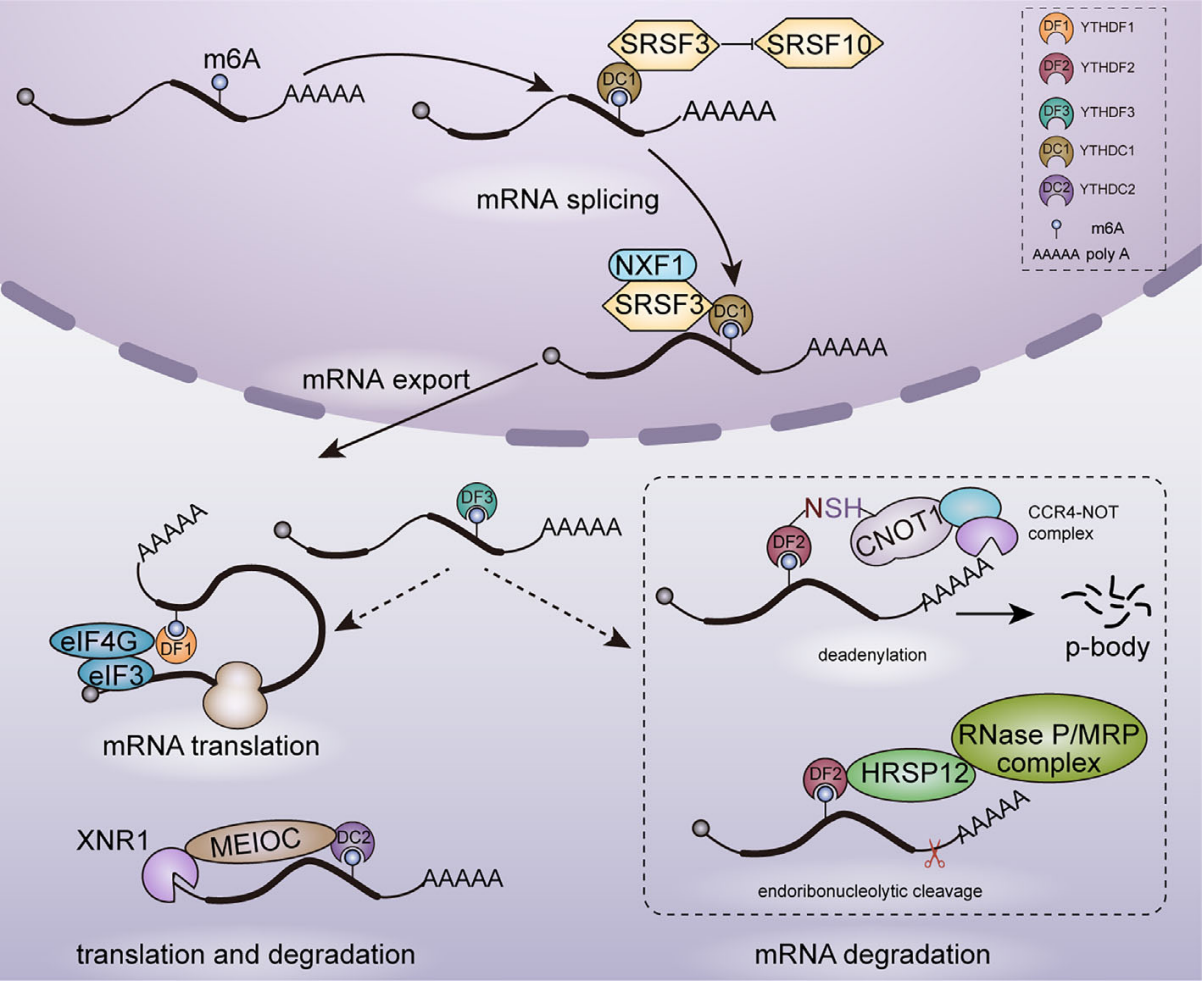
Functions of YTH family proteins in mRNA metabolism. In nucleus, YTHDC1 participates in mRNA splicing and nuclear export. In cytoplasm, YTHDF1/2/3, YTHDC2 are involved in mRNAs translation and degradation.

mRNA metabolism refers to the entire mRNA life from birth to death that includes transcription, mRNA processing, mRNA nuclear exportation, mRNA translation and mRNA degradation. YTH family proteins play an essential role in regulating cellular fates of m6A-modified mRNAs. In 5 human YTH family proteins, YTHDC1 is the only nuclear protein involved in transcription (31), mRNA splicing (32) and nuclear export (33), whereas YTHDF1 (34, 35), YTHDF2 (36, 37), YTHDF3 (34, 38) and YTHDC2 (39) are cytoplasmic m6A readers mainly involved in mRNA translation and degradation. As shown in **Figure 1**, YTH family proteins are almost involved in every step of mRNA metabolism.

### Regulation in mRNA Splicing and Nuclear Export

YTHDC1 is located in the nucleus and forms a novel compartment called YT bodies, which are adjacent to RNA processing speckles enriching mRNA splicing factors (28). This nuclear localization is consistent with that YTHDC1 interacts with several splicing regulators including SRSF3 (40), SAM68 (41) and SC35 (42). Role of YTHDC1 in regulating mRNA splicing was established in *Drosophila* sex determination and mouse embryogenesis. In *Drosophila*, YTHDC1 decoded m6A in the spliced intron of sex determination factor Sex lethal (Sxl) and facilitated the alternative splicing of Sxl pre-mRNA, which determines female physiognomy (43). In mouse oocytes, loss of YTHDC1 led to extensive alternative polyadenylation and massive defects in alternative splicing, which hindered oocyte maturation (33). Mechanically, YTHDC1 regulated mRNA splicing by recruiting pre-mRNA splicing factor SRSF3 to promote exon inclusion whiles blocking binding of SRSF10, which facilitated exon skipping (44).

Despite the essential role in alternative splicing, YTHDC1 was also reported to promote the export of methylated mRNA from nucleus to cytoplasm in HeLa cells. This process relied on the interaction with the splicing factor SRSF3 which functions as a key adaptor in nuclear RNA export factor 1 (NXF1) -dependent nuclear export pathway (33).

### Regulation in mRNA Translation

Knockdown of YTHDF1 in HeLa cells led to reduced translation efficiency of its targeted transcripts and YTHDF1 was found to promote ribosome loading of targeted mRNAs, indicating the role of YTHDF1 in enhancing mRNA translation. With METTL3 depleted, the translation efficiency of YTHDF1 targeted transcripts was decreased completely. It confirmed that YTHDF1 promoted translation efficiency in an m6A-dependent manner. However, the underlying mechanism of YTHDF1 promoting translation efficiency is still unclear currently. It may rely on the interaction with eukaryotic initiation factor 3 (eIF3) and eIF4G-mediated loop formation (35). YTHDF3 shares numerous similar targets with YTHDF1 and YTHDF2: 58% of YTHDF3 targets are also recognized by YTHDF1 and 60% by YTHDF2, which indicates a potential coordination on common targets among YTHDF proteins. YTHDF3 was found to promote the translation of targeted mRNAs through interacting with YTHDF1 (34).

In YTHDC2–/– mouse testes, translation efficiency of YTHDC2 targets structural maintenance of chromosomes 3 (Smc3) and centrosomal protein 76 (Cep76) showed a significant decrease, whereas mRNA abundance showed an increase. Furthermore, YTHDC2 was observed in the 40–80S ribosome fraction, indicating that YTHDC2 may enhance translation efficiency by interacting with cellular machinery involved in translation initiation (39).

### Regulation in mRNA Degradation

mRNA degradation plays key roles in gene expression regulation and mRNA quality control. YTHDF2 is the main m6A reader protein to promote the decay of m6A-containing mRNA (2, 36, 45). YTHDF2 selectively recognized m6A-containing mRNA through C-terminal of YTH domain, whereas its N-terminal region localized the YTHDF2-m6A-mRNA complex from the translatable pool to mRNA decay sites such as processing bodies (P-bodies) for mRNA degradation (43). Furthermore, YTHDF2 triggered deadenylation and degradation of m6A-containing mRNAs by recruiting the carbon catabolite repression 4–negative on TATA-less (CCR4–NOT) deadenylase complex, with N-YTHDF2 interacting with the superfamily homology (SH) domain of CCR4–NOT transcription complex subunit 1 (CNOT1) (36). Despite CCR4-NOT complex-mediated deadenylation pathway, m6A RNAs bound by YTHDF2 can be degraded through another RNase P/MRP-mediated endoribonucleolytic pathway. An adaptor protein heat-responsive protein 12 (HRSP12) bridged YTHDF2 and RNase P/MRP, which aroused endoribonucleolytic cleavage of YTHDF2-binding m6A RNAs (45). Moreover, YTHDF3 was identified to accelerate the degradation of mRNAs through interacting with YTHDF2 (34).

In addition to YTHDF proteins, YTHDC2 also plays a role in affecting mRNA stability and promoting mRNA degradation. In a study on regulation of m6A on hepatic drug-metabolizing enzyme, YTHDC2 was identified to promote cytochrome P450 family 2 subfamily C member 8 (CYP2C8) mRNA degradation by recognizing the m6A-modified CYP2C8 mRNA and negatively regulated CYP2C8 expression (46). YTHDC2 is highly expressed in mouse testes and YTHDC2 plays an important role in facilitating proper spermatocyte development (39). YTHDC2 has ATPase and a 3′-5′ RNA helicase activity. It interacted with the meiosis-specific MEIOC protein as well as with 5′-3′ exoribonuclease XRN1 *via* the Ankyrin repeat insertion that is located within the helicase domain of YTHDC2 and then degraded m6A-containing transcripts (29). In contrast to YTHDF2, YTHDC2 regulates RNA stability in an RNA-independent manner through increasing the local concentration of the RNA decay machineries.

### Regulation in RNA Phase Separation

The main mechanism by which YTHDF proteins target m6A-containing mRNAs to exact functions in various biological processes is through liquid-liquid phase separation (LLPS). Structurally, besides YTH domain at C-termini, all three YTHDF proteins have a low complexity domain at their N-termini, which seems to be Prion-like domain and has the potential to undergo phase separation. YTHDF proteins bound m6A-containing mRNA to undergo phase separation and formed RNA-protein droplets. These RNA-protein droplets partitioned into different endogenous phase-separated compartments, such as P-bodies, stress granules and other RNA-protein assemblies, where mRNAs could be stored or degraded (47–49). In addition, YTHDF proteins play an important role in promoting stress granule (SG) formation. They functioned as SG shell proteins and promoted SG formation by bringing together multiple SG core clusters to form large granules under oxidative stress (50).

### Regulation in Transcription

Knockout of METTL3 or YTHDC1 in mouse embryonic stem cells was found to increase chromatin accessibility and activate transcription in an m6A-dependent manner. Mechanistically, nuclear reader YTHDC1 recognized m6A-modified chromosome-associated regulatory RNAs (carRNAs) deposited by METTL3 and promoted the decay of these RNAs through the NEXT-mediated nuclear degradation (31). Nuclear exosome targeting (NEXT) complex contains hMTR4, the Zn-knuckle protein ZCCHC8 and the putative RNA binding protein RBM7 (51). YTHDC1 was involved in nuclear degradation by interacting with the NEXT components RBM7 and ZCCHC8. However, whether these carRNAs play a role in various types of cancers warrants further studies. Currently, studies on m6A function mainly focus on post-transcription regulation, more precise mechanisms of m6A in regulating transcription are needed to be further investigated.

### Dispute on Regulation of YTHDF Proteins in mRNA Metabolism

According to the prevailing mode for functions of YTHDF proteins in regulating mRNA metabolism, YTHDF1 enhances translation efficiency; YTHDF2 facilitates mRNA degradation; and YTHDF3 promotes translation and degradation by interacting with YTHDF1 and YTHDF2 (35, 43). Each YTHDF paralog controls various physiologic processes and diseases by targeting specific cohorts of m6A-containing mRNAs. However, other studies suggested that these three YTHDF proteins may have similar roles in mRNA degradation by recruiting the main mRNA deadenylation complex CCR4–NOT on m6A-containing mRNA (36, 52). In a recent study, Zaccara et al. proposed a novel unified model that all YTHDF paralogs bound to all m6A sites in a similar manner. The main effect of three YTHDF proteins was to facilitate the degradation of the same subset of mRNAs in a redundant manner, rather than enhance translation as previous studies suggested (53). Whether these three YTHDF proteins are functionally redundant and whether YTHDF proteins enhance mRNA translation are still disputed. Zaccara et al. thought that links between YTHDF proteins and mRNA translation presented by previous studies were affected by bioinformatic and technical issues, which may lead to the incorrect view that a major function of YTHDF proteins was to promote translation. So more independent studies with careful experimental design are needed to answer these questions in the future. In addition, interplay between these YTHDF proteins in impacting mRNA translation or degradation, and their interacting proteins involved in different cellular processes still warrant further research.

## Roles of YTH Family Proteins in Cancers

**Figure 2:**
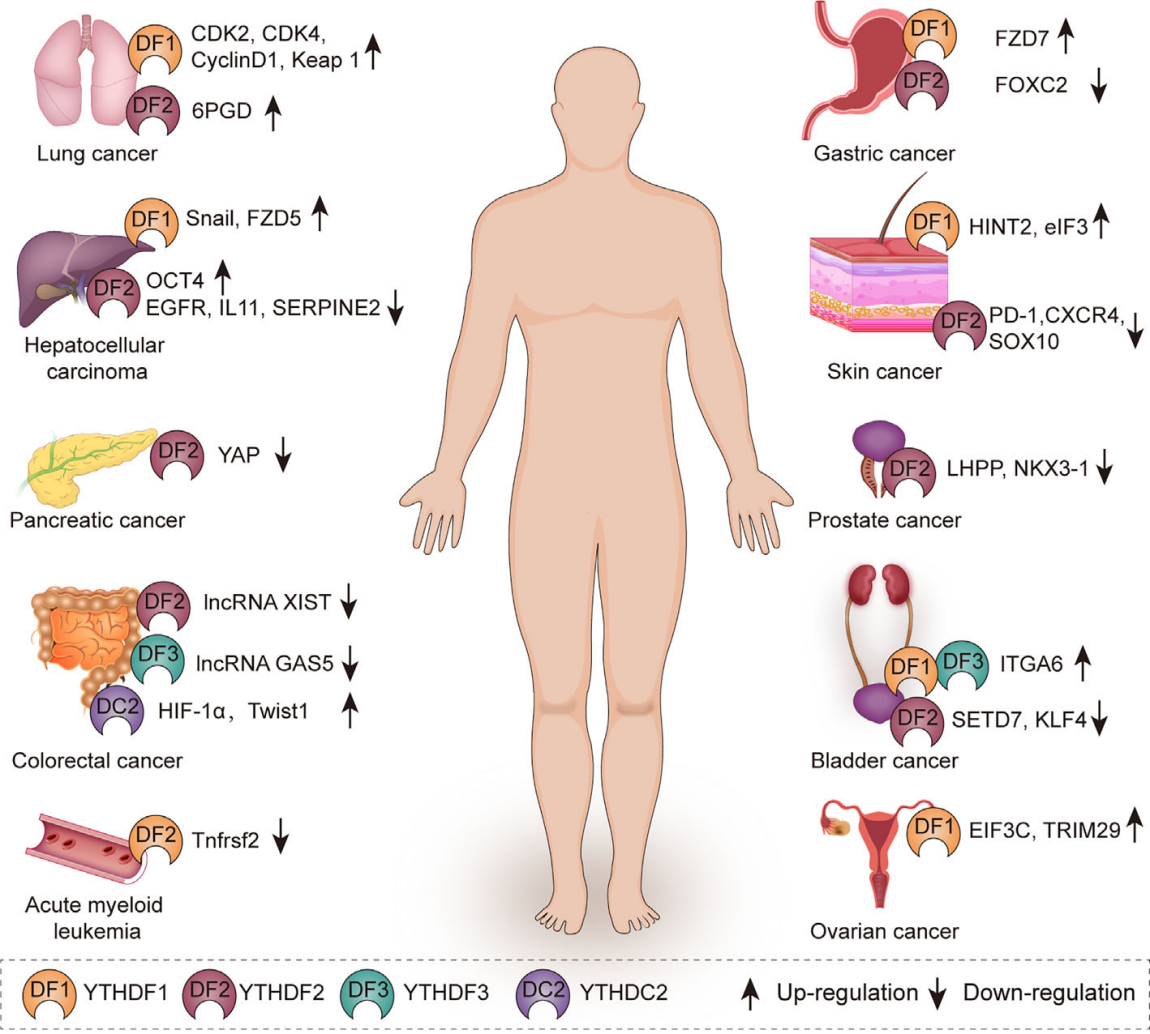
The role of YTH family proteins in human cancers. YTH family proteins are associated with various kinds of cancers including lung cancer, hepatocellular carcinoma, pancreatic cancer, colorectal cancer, acute myeloid leukemia, gastric cancer, skin cancer, prostate cancer, bladder cancer, and ovarian cancer.

YTH family proteins play important roles in physiological processes through mediating metabolic process of targeted RNAs (54), such as sex determination (32, 55, 56), oocyte development (57), spermatogenesis (39), stem cell maintenance and differentiation (55, 56), and stress response (55, 56, 58). Recently, the increasing evidences have shown that YTH family proteins exert effects on multiple human diseases. In this part, we focus on the roles of these YTH family proteins in various cancers (**Figure 2**, **Table 1**).

**Table 1:** Multiple functions exerted by YTH family proteins in various cancers.

Molecule	Cancer type	Target	Effect on target	Role in cancer	Reference
YTHDF1	HCC	Snail	Translation	Promote EMT of HCC cells	(59)
	HCC	FZD5	Translation	Promote cell proliferation and metastasis	(60)
	Gastric cancer	FZD7	Translation	Promote cell proliferation	(61)
	Lung cancer	CDK2, CDK4, cyclin D1	Translation	Promote cell proliferation	(62)
	Ovarian cancer	EIF3C	Translation	Promote cell proliferation and metastasis	(63)
	Ovarian cancer	TRIM29	Translation	Enhance CSC-like characteristics	(64)
	Ocular melanoma	HINT2	Translation	Inhibit cell proliferation and migration	(65)
	MCC	eIF3	Translation	Promote cell proliferation	(66)
YTHDF2	HCC	OCT4	Translation	Promote CSC phenotype and metastasis	(67)
	HCC	EGFR	Degradation	Inhibit cell proliferation	(68)
	HCC	IL11, SERPINE2	Degradation	Inhibit inflammation, vascular remodeling and metastasis	(69)
	Pancreatic cancer	YAP	Expression	Promote cell proliferation but inhibit migration and invasion	(70)
	Gastric cancer	FOXC2	Expression	Inhibit cell proliferation, invasion and migration	(71)
	CRC	XIST	Degradation	Inhibit cell proliferation and metastasis	(72)
	Lung cancer	6PGD	Translation	Promote cell proliferation	(22)
	AML	Tnfrsf2	Stability	Promote LSC development and AML initiation	(73)
	Bladder cancer	SETD7, KLF4	Degradation	Promotes cell migration	(74)
	Prostate cancer	LHPP, NKX3–1	Degradation	Promote cell proliferation and migration	(75)
	Melanoma	PD-1, CXCR4, SOX10	Degradation	Inhibit cell proliferation and migration	(76)
YTHDF3	CRC	LncRNA GAS5	Degradation	Promote CRC progression	(77)
YTHDF1/3	Bladder cancer	ITGA6	Translation	Promote cell proliferation and migration	(78)
YTHDC2	CRC	HIF-1α,Twist1	Translation	Promote cell metastasis	(79)

HCC, hepatocellular carcinoma; CRC, colorectal cancer; MCC, Merkel cell carcinoma; AML, acute myeloid leukemia; EMT, epithelial mesenchymal transition; CSC, cancer stem cell; LSC, leukemic stem cell.

### Hepatocellular Carcinoma (HCC)

Hepatocellular carcinoma is one of the most aggressive malignancies, which ranks as the fourth leading cause of cancer-related deaths worldwide. In 188 clinical HCC tissues, immunohistochemistry staining (IHC) results showed that the positive rate of YTHDF2 was 35.6% and nearly 83.9% in grade III HCC tissues, which revealed that YTHDF2 was closely associated with HCC (80). YTHDF2 was found to promote the liver cancer stem cell phenotype and cancer metastasis by enhancing OCT4 mRNA translation in a m6A-dependent manner (67). On the contrary, YTHDF2 was reported to function as a tumor suppressor in other studies, YTHDF2 inhibited the proliferation and growth of HCC cells *via* promoting the degradation of epidermal growth factor receptor (EGFR) mRNA, which is the upstream of the ERK/MAPK pathway (68). Moreover, silence of YTHDF2 escalated inflammation, vascular remodeling and metastasis of HCC. Mechanistically, YTHDF2 plays a suppressive role in HCC through promoting the degradation of inflammatory cytokines interleukin 11 (IL11) and serpin family E member 2 (SERPINE2) mRNAs (69).

YTHDF1 acts as an oncogene in HCC. YTHDF1 was overexpressed and positively correlated with pathology stage in HCC patients by analyzing TCGA data. Up-regulation of YTHDF1 was associated with poor prognosis in HCC patients (81, 82). Mechanistically, YTHDF1 promotes the epithelial mesenchymal transition (EMT) of HCC cells *via* promoting the translation of snail family transcriptional repressor 1 (Snail) mRNA (59). Similarly, YTHDF1 was also observed to promote HCC cell proliferation and metastasis by promoting the translational output of frizzled5 (FZD5) mRNA in an m6A-dependent manner and acts as an oncogene through activating the WNT/β-catenin pathway (60). Accordingly, YTHDF1 can be a potential biomarker for HCC diagnosis and prognosis.

### Pancreatic Cancer

Pancreatic cancer is associated with extremely poor prognosis and remains a highly lethal disease due to difficulties in early diagnosis and metastasis. Interestingly, YTHDF2 plays the dual role in pancreatic cancer progression. It promoted the proliferation but suppressed migration and invasion of pancreatic cancer cells, which is so called “migration-proliferation dichotomy”. Mechanistically, silence of YTHDF2 inhibited proliferation through Akt/GSK3b/CyclinD1 pathway and promoted EMT *via* up-regulation of yes-associated protein (YAP), YAP serves as the core components of the Hippo pathway, which can promote the proliferation and survival of epithelial cells (70).

### Gastric Cancer

Gastric cancer is the fifth most common cancer and ranks third in cancer-related death worldwide. YTHDF1 acts as an oncogene in gastric cancer. High expression of YTHDF1 was associated with poor overall survival in gastric cancer patients. Suppression of YTHDF1 inhibited the proliferation and tumorigenesis of gastric cancer cells. Mechanistically, YTHDF1 recognizes m6A-modified frizzled7 (FZD7) mRNA and accelerates its translation. FZD7 is a key Wnt receptor and increased expression of FZD7 leads to hyper-activation of the Wnt/β-catenin pathway and promotion of gastric carcinogenesis (61). In addition, YTHDF2 is down-regulated in gastric cancer tissues and cells. Overexpression of YTHDF2 significantly inhibited the proliferation, invasion and migration of gastric cancer cells by negatively regulating forkhead box C2 (FOXC2), which acts as an oncogene in various cancers such as nasopharyngeal cancer, colorectal cancer and triple negative breast cancer (71, 83–85).

### Colorectal Cancer

YTHDF1 acts as an oncogene in colorectal cancer (CRC) and is associated with poor prognosis in overall survival. Down-regulation of YTHDF1 inhibited CRC proliferation and increased sensitivity to the exposure of fluorouracil and oxaliplatin. Moreover, YTHDF1 is transcriptionally regulated by a well-known oncogenic transcription factor c-Myc in CRC (86). Mechanistically, knockdown of YTHDF1 significantly suppressed Wnt/β-catenin pathway activity in CRC. Wnt/β-catenin pathway is one of the best-characterized cancer drivers that can promote cancer progression and chemoresistance in various cancers (87). YTHDF1 plays an essential oncogenic role in CRC and is expected to be a therapeutic target for CRC.

YTHDC2 promotes the metastasis of colon cancer cells by facilitating the translation of hypoxia inducible factor 1 subunit alpha (HIF-1α) and twist family BHLH transcription factor 1 (Twist1) mRNA during hypoxia (79). In addition, METTL14 inhibits the malignancy of CRC by suppressing oncogenic long non-coding RNA XIST. YTHDF2 recognizes m6A-modified XIST and accelerated the decay of XIST (72). LncRNA GAS5 inhibits CRC progression *via* triggering phosphorylation and ubiquitin-mediated degradation of YAP. YTHDF3 is a target of YAP and furthermore plays a key role in YAP signaling by promoting m6A-modified lncRNA GAS5 decay (77).

### Lung Cancer

Lung cancer is the leading cause of cancer-related death worldwide. YTHDF proteins play different roles in lung cancer progression by controlling the metabolism of different targets in a m6A-dependent manner. YTHDF2 is up-regulated in lung cancer tissues and functions as an oncogene in lung cancer. YTHDF2 promotes the proliferation of lung cancer cells and facilitates the pentose phosphate pathway (PPP) flux, which is critical in regulating cancer cell growth by supplying cells with ribose-5-phosphate and NADPH. Mechanistically, YTHDF2 directly binds to m6A-modified 6-phosphogluconate dehydrogenase (6PGD) mRNA and promotes 6PGD mRNA translation. 6PGD is upregulated in lung cancer and elevated expression of 6PGD can promote lung cancer cell proliferation (22).

YTHDF1 promotes the proliferation of non-small cell lung cancer (NSCLC) cells by enhancing the translational efficiency of cell cycle regulators CDK2, CDK4 and cyclinD1. However, the clinical correlation analysis showed the adverse result. YTHDF1 high expression correlated with better clinical outcome in NSCLC and depletion of YTHDF1 rendered NSCLC cells resistant to cisplatin (DDP) treatment. Mechanistically, depletion of YTHDF1 can inhibit the translational efficiency of m6A-modified Keap1 mRNA in an oxidative stress state induced by DDP, and activated the antioxidant ROS clearance system (Nrf2-AKR1C1) in turn, leading to DDP resistance and a worse clinical outcome for NSCLC patients (62). According to above findings, it may provide a potential strategy to improve the clinical outcome of YTHDF1 low expressing NSCLC patients by using AKR1C1 specific inhibitors together with platinum based chemotherapy.

### Acute Myeloid Leukemia (AML)

AML is a disorder characterized by a clonal proliferation derived from primitive hematopoietic stem cells (HSCs) or progenitor cells, resulting in blockade of myeloid differentiation and generation of self-renewing leukemic stem cells (LSCs). It is still a challenge to eliminate cancer stem cells while preserving hematopoiesis in leukemia treatment. YTHDF2 is up-regulated in AML and plays an essential role in leukemic stem cell (LSC) development and AML initiation by decreasing the half-life of tumor necrosis factor receptor TNF receptor superfamily member 2 (Tnfrsf2), which contributes to the overall integrity of LSC function. More importantly, YTHDF2 deficiency cannot derail hematopoiesis and can enhance HSC activity. So YTHDF2 is a unique therapeutic target in AML whose depletion selectively inhibits LSCs while accelerating HSC expansion (73).

### Ovarian Cancer

Ovarian cancer ranks as the fifth leading cause of cancer-related death in women worldwide and has the highest mortality rate among gynecological cancers due to poor prognosis and high relapse rate. YTHDF1 is frequently overexpressed in ovarian cancer and up-regulation of YTHDF1 is associated with the adverse prognosis of ovarian cancer patients. Silence of YTHDF1 inhibited the growth and metastasis of ovarian cancer *in vitro* and *in vivo*. Mechanistically, YTHDF1 facilitates the malignancy by binding to m6A-modified eukaryotic translation initiation factor 3 subunit C (EIF3C) mRNA, which is a subunit of the protein translation initiation factor EIF3, and promotes the translation of EIF3C mRNA (63). In addition, cancer stem cells (CSCs) are a population of cells with stem-like characteristics that are able to cause chemoresistance and recurrence. YTHDF1 was found to enhance the CSC-like characteristics of the cisplatin-resistant ovarian cancer cells by binding to m6A-modified TRIM29 mRNA and promoting its translation (64). Thus, targeting YTHDF1 is expected to be a promising candidate for ovarian cancer therapy.

### Bladder Cancer

In bladder cancer, three YTHDF proteins all act as oncogenes. YTHDF1/YTHDF3 promotes the tumor growth and progression by recognizing m6A-modified integrin subunit alpha 6 (ITGA6) mRNA and promoting its translation (78). YTHDF2 facilitates tumorigenesis through accelerating the degradation of tumor suppressors set domain containing 7 (SETD7) mRNA and Kruppel like actor 4 (KLF4) mRNA in bladder cancer (74).

### Prostate Cancer

YTHDF2 was found frequently up-regulated in prostate cancer through immuno-histochemical (IHC) staining and chromogenic *in situ* hybridization (CISH). Knockdown of YTHDF2 inhibited the proliferation and migration of prostate cancer cells. MiR-493-3p was identified to be an upstream factor of YTHDF2, which suppressed prostate cancer by targeting YTHDF2 (88). Another study had the similar results, YTHDF2 mediated the degradation of tumor suppressors LHPP and NKX3–1 mRNA and indirectly induced AKT phosphorylation to promote prostate cancer progression in an m6A-dependent manner (75). Above results suggest that YTHDF2 acts as an oncogene in prostate cancer and YTHDF2 is expected to be a potential biomarker for diagnosis or targeted therapy of prostate cancer.

### Skin Cancer

Melanoma is one of the most aggressive malignant skin tumors and its incidence has been increasing worldwide in recent decades. In melanoma, YTHDF2 suppresses the proliferation and migration of melanoma cells by promoting the decay of protumorigenic melanoma cell-intrinsic genes such as PD-1 (PDCD1), CXCR4 and SOX10 (76). In addition, YTHDF1 inhibits the growth and migration of ocular melanoma cells *via* facilitating the translation of histidine triad nucleotide binding protein 2 (HINT2) (65). Merkel cell carcinoma (MCC) is a kind of highly malignant skin cancer, of which 80% cases are mainly caused by the Merkel cell polyomavirus (MCPyV) (89). YTHDF1 is highly expressed in MCC, silence of YTHDF1 down-regulated the translational initiation factor eIF3, leading to the reduction of proliferative and clonogenic capacity in MCC cells (66).

## Potential Application of YTH Family Proteins in Cancer Therapy

YTH family proteins serve as the potential therapeutic and prognostic targets in various cancers. For example, YTHDF2 is a critical regulator for acute myeloid leukemia (AML) initiation and propagation. Deficiency of YTHDF2 can limit leukemic stem cells activity while enhancing hematopoietic stem cells expansion and myeloid reconstitution. Thus, inhibition of YTHDF2 is expected to be a potential therapeutic strategy for AML treatment (73). In ovarian cancer, YTHDF1 acts as an oncogene. Up-regulation of YTHDF1 is associated with the poor prognosis of ovarian cancer patients and knockdown of YTHDF1 can inhibit the stem cell-like features of cisplatin-resistant ovarian cancer cells. YTHDF1 may have strong potential as a therapeutic target for ovarian cancer (63, 64).

Nowadays, despite surgery, chemotherapy and radiotherapy, immunotherapy has become a promising method in cancer treatment. Immune check-point therapy based on cytotoxic T lymphocyte-associated antigen 4 (CTLA4), programmed death-1 (PD-1), and programmed death ligand-1 (PD-L1) inhibitors has a good effect in non–small-cell lung carcinoma and melanoma (90, 91). YTHDF1 deficiency was found to exert antitumor function by enhancing immunosurveillance. Loss of YTHDF1 facilitated the cross-presentation of tumor antigens on dendritic cells (DCs) and increased cross-priming of CD8^+^ T cells. Mechanistically, YTHDF1 recognizes m6A-modified transcripts encoding lysosomal cathepsins in DCs and promotes their translation, which inhibits the cross-presentation of tumor neoantigens to achieve immune escape. In addition, YTHDF1 deficiency improves the therapeutic outcome of immune checkpoint inhibitor, which blocks the T cell inhibitor receptor PD1. Combined with immune checkpoint blockade, YTHDF1 can be a potential new target in cancer immunotherapy (92).

## Conclusion and Perspectives

YTH family proteins, as the main m6A reader proteins, participate in tumorigenesis, proliferation, invasion and metastasis of various cancers through regulating almost the entire process of targeted RNAs metabolism. According to the prevailing mode, the nuclear reader YTHDC1 is involved in mRNA splicing and nuclear export. YTHDF2 promotes mRNA degradation; YTHDF1 enhances translation; and YTHDF3 both accelerates translation and degradation through interacting with YTHDF1 and YTHDF2. However, whether three YTHDF proteins have distinct or redundant cellular functions has been disputed in recent studies. Considering that the expression of YTHDF2 is more highly than YTHDF1 and YTHDF3, effects of YTHDF1 or YTHDF3 deficiency may not be similar to YTHDF2 deficiency due to compensation. So silencing all three YTHDF proteins and then expressing one YTHDF protein exogenously may be a good way to detect their redundant functions. These disputes need to be settled through more careful experimental design in further studies.

YTH family proteins mainly act as oncogenes in different types of cancer with few exceptions. For example, YTHDF2 inhibits the progression of gastric cancer and melanoma. And YTHDF1 plays a suppressive role in ocular melanoma. The different roles of YTH family proteins act in various cancers may depend on their specific recognition of different m6A-mdified transcripts, which act as oncogenes or tumor suppressors. It is essential to identify more m6A-modified transcripts recognized by YTH family proteins, verify roles of these targets acted in cancer progression, and clarify the mechanism by which YTH family proteins achieve selectivity toward certain m6A-modified transcripts. In addition, different researchers presented the opposite role of the same YTH family protein acted even in the same cancer. Therefore, more large-scale and multi-center researches are needed to explore the functions and underlying mechanisms of YTH family proteins in various cancers, which provides basis for precise cancer treatment.

Studies on regulation of YTH family proteins in cancer progression are few. Recently, an additional function of non-coding RNAs in the control of YTH family proteins has been reported. In hepatocellular carcinoma (HCC), miR-145 modulates m6A levels by targeting the 3′-UTR of YTHDF2 mRNA and inhibits the progression of HCC cells (93). In prostate cancer, miR-493-3p is an upstream factor of YTHDF2 and exerts anti-tumor effects by targeting YTHDF2 (88). The profound mechanism by which interplay between YTH family proteins and non-coding RNA impacts cancer development needs to be further studied. In addition, post-translational modification plays an important role in regulating YTHDF family proteins. The SUMOylation of YTHDF2 at the major site of K571 significantly increases its binding affinity of m6A-modified mRNAs and results in deregulated gene expressions which accounts for cancer progression (94). Is there any other post-translational modification to regulate YTH family proteins and impact cancer progression? Whether there are external or internal stimuli leading to alteration of post-translational modification? These questions are worthy of further exploration.

The essential roles of m6A observed in various types of cancers suggest that they are potential therapeutic targets for cancer therapy. It has aroused great interest to develop small-molecule inhibitors targeting m6A writers or erasers. For example, meclofenamic acid (MA) and R-2-hydroxyglutarate (R-2HG), which can inhibit the activity of FTO, are applied to treat leukemia and glioma (95, 96). However, another approach that can be explored is to target YTH family proteins pharmacologically. Considering that YTH domain has a unique aromatic cage structure, the aromatic cage forms a hydrophobic binding pocket that is crucial for the specific recognition and binding of m6A. This site may be suitable for developing small molecule inhibitors that can compete with m6A-modified transcripts and counteract the effects of YTH family proteins. Besides small molecule inhibitors that target YTH family proteins directly, PROTAC (proteolysis targeting chimera)-based inhibitors which can selectively decay dysregulated m6A reader proteins may be a feasible method for cancer therapy. Development of small molecule inhibitors for targeting YTH family proteins could lead to a new way of cancer therapy in the future.

## Author Contributions

QD and J-FW designed the study. X-YD, LS, and ZL wrote the manuscript. H-YY revised the manuscript. All authors contributed to the article and approved the submitted version.

## Funding

This work was supported by the National Natural Science Foundation of China (81972486, 81802748, 81802644), the Key Medical Talents of Jiangsu Province (ZDRCA2016029) and ‘333’ High-level Talents Training Project of Jiangsu Province (BRA2016505). Besides, the work was also funded by the International Cooperation Project of Jiangsu Provincial Science and Technology Department (BZ2018054) and the Priority Academic Program Development of Jiangsu Higher Education Institutions (PAPD).

## Conflict of Interest

The authors declare that the research was conducted in the absence of any commercial or financial relationships that could be construed as a potential conflict of interest.
